# Oncogenic *HER2* fusions in gastric cancer

**DOI:** 10.1186/s12967-015-0476-2

**Published:** 2015-04-11

**Authors:** De-Hua Yu, Lili Tang, Hua Dong, Zhengwei Dong, Lianhai Zhang, Jiangang Fu, Xinying Su, Tianwei Zhang, Haihua Fu, Lu Han, Liang Xie, Hao Chen, Ziliang Qian, Guanshan Zhu, Jia Wang, Qingqing Ye, Jingchuan Zhang, Xiaolu Yin, Xiaolin Zhang, Jiafu Ji, Qunsheng Ji

**Affiliations:** Innovation Center China, Asia & Emerging Market iMed, AstraZeneca Innovation Medicines and Early Development, 199 Liangjing Road, Zhangjiang Hi-Tech Park, Shanghai, 201203 China; Key laboratory of Carcinogenesis and Translational Research (Ministry of Education), Department of Surgery, Peking University Cancer Hospital and Institute, Beijing, China; Department of General Surgery, Renji Hospital, School of Medicine, Shanghai Jiao Tong University, Shanghai, China; Current mailing address: WuXi AppTec, 288 Fute Zhong Road, Waigaoqiao, China (Shanghai) Pilot Free Trade Zone, Shanghai, 200131 China

**Keywords:** HER2, Fusion-gene, Gastric cancer, Trastuzumab, Lapatinib

## Abstract

**Background:**

Genetic amplification of *HER2* drives tumorigenesis and cancer progression in a subset of patients with gastric cancer (GC), and treatment with trastuzumab, a humanized HER2-neutralizing antibody, improves the overall survival rate of HER2-positive patients. However, a considerable portion of the patients does not respond to trastuzumab and the molecular mechanisms underlying the intrinsic resistance to anti-HER2 therapy in GC is not fully understood.

**Methods:**

We performed whole-transcriptome sequencing on 21 HER2-positive tumor specimens from Chinese GC patients. Whole genome sequencing was performed on the three samples with *HER2* fusion to discover the DNA integration structure. A multicolor FISH assay for *HER2* split screening was conducted to confirm *HER2* fusion and IHC (HercepTest™) was used to detect the membranous expression of *HER2*. Fusion cDNA were transfected into NIH/3T3 cells and generate stable cell line by lentivirus. The expression of exogenous *HER2* fusion proteins and *pHER2* were examined by western blot analysis. *In vitro* efficacy studies were also conducted by PD assay and softagar assay in cell line expression wild type and fusion *HER2*. T-DM1 was used to assess its binding to NIH/*3T3* cells ectopically expressing wild-type and fusion HER2. Finally, the anti-tumor efficacy of trastuzumab was tested in NIH/3 T3 xenografts expressing the *HER2* fusion variants.

**Results:**

We identified three new *HER2* fusions with *ZNF207*, *MDK*, or *NOS2* in 21 *HER2-*amplified GC samples (14%; 3/21). Two of the fusions, *ZNF207-HER2*, and *MDK-HER2*, which are oncogenic, lead to aberrant activation of HER2 kinase. Treatment with trastuzumab inhibited tumor growth significantly in xenografts expressing MDK-HER2 fusion. In contrast, trastuzumab had no effect on the growth of xenografts expressing ZNF207-HER2 fusion, due to its inability to bind to trastuzumab.

**Conclusions:**

Our results provide the molecular basis of a novel resistance mechanism to trastuzumab-based anti-HER2 therapy, supporting additional molecule stratification within HER2-positive GC patients for more effective therapy options.

**Electronic supplementary material:**

The online version of this article (doi:10.1186/s12967-015-0476-2) contains supplementary material, which is available to authorized users.

## Background

Gastric cancer (GC), as the second leading cause of cancer deaths worldwide, accounted for 989,600 new cases and 738,000 deaths globally in 2011 [1]; more than 50% of GC cases occur in Eastern Asia [2]. The conventional treatments for GC include surgery, radiotherapy and chemotherapy [3], which have limited efficacy because most GC patients are in the advanced stages when diagnosed; the five-year survival rate for patients with stage III/IV GC is around 10% [4]. Trastuzumab, a neutralization antibody of HER2, was recently approved for the treatment of a subset of advanced GC patients whose tumors are clinically defined as HER2-positive.

*HER2* gene amplification was initially discovered as an oncogene in breast cancer (BC), which led to the development of HER2-targeted therapeutics for treating HER2-positive BC [5]. These drugs include trastuzumab; lapatinib, a small-molecular inhibitor of HER2 kinase; pertuzumab, an antibody-blocking heterodimerization of HER2 with HER3; and trastuzumab emtansine (T-DM1), which is trastuzumab conjugated with the antimitotic agent emtansine (DM1). The clinical application of these targeted agents dramatically changed the landscape of BC therapy and exemplified a new era of personalized medicine associated with companion molecular diagnosis for patient selection [6-8]. In addition to BC, *HER2* amplification and overexpression was also found in about 20% of GC patients [9]. The anti-tumor activity of trastuzumab as a single agent or in combination with cytotoxic agents has been demonstrated in several HER2-positive human GC cell lines *in vitro* and in GC xenografts *in vivo* [10-12]. The preclinical efficacy translated into positive clinical trials in which a survival improvement was achieved in HER2-positive metastatic GC patients treated with trastuzumab plus cytotoxic agents [9,13]. These results led to the approval of trastuzumab as the first molecular targeted therapy for treating GC.

Despite the clinical benefits of trastuzumab in the treatment of patients with HER2-positive GC or BC [13,14], approximately 30-40% of HER2-positive tumors are insensitive to the treatment. Significant efforts to understand the resistance to anti-HER2 therapy in BC cases have recently been made, resulting in a diverse array of resistance mechanisms and clinical strategies to overcome the resistance [15]. However, there is little understanding of the resistance mechanism to anti-HER2 therapy in GC. Therefore, we used a next-generation sequencing (NGS) approach to elucidate molecular insights in HER2-positive GC. In this study, for the first time, we report three *HER2* gene fusions in HER2-positive GC in Chinese patients, and we characterize their oncogenic properties and sensitivity to anti-HER2 agents.

## Methods

### Human primary tumor samples

Specimens were collected during surgery from Chinese GC patients with postoperative pathological confirmation. The study was carried out at Peking University Cancer Hospital and Institute, and Shanghai Renji Hospital (2007 ~ 2010). Written informed consent was provided by each patient, and the study was approved by the ethics committees of the hospitals.

### RNA-seq for transcriptome analysis

Total RNA was extracted using TRIzol (Life Technologies). All RNA samples showed RNA integrity numbers >7 (Agilent 2100 bioanalyzer).

Total RNA quality and concentration was measured using an RNA Pico chip on a Bioanalyzer 2100 (Agilent). Normalized starting quantities of total RNA were used to prepare Illumina sequencing libraries with a TruSeq™ RNA sample preparation kit (Illumina). The library preparation was performed according to the manufacturer’s instructions. The cDNA libraries were placed on an Illumina c-Bot for paired-end (PE) cluster generation, according to the protocol outlined in the Illumina HiSeq Analysis User Guide. The template cDNA libraries (1.5 μg) were hybridized to a flow cell, amplified, linearized, and denatured to create a flow cell with ssDNA ready for sequencing. Each flow cell was sequenced on an Illumina HiSeq2000 sequencing system. After a 100-cycle PE sequencing run, the bases and quality values were generated for each read with the current Illumina pipeline.

### Detection of fusion transcripts

We sequenced each tumor sample up to an average of about 150× coverage. Fusion transcripts were detected using FusionMap software [16]. Fusions supported by at least three reads were selected as candidates and subjected to RT-PCR and Sanger sequencing confirmation.

### Quantification of mRNA expression level

Human gene expression quantification was measured according to sequenced fragments (reads) per kilobase of exon per million fragments mapped to the human genome (FPKM):

$$ FPKM=\frac{10^9*N}{L*R} $$

N: number of reads mapped in gene

L: gene length (bp) (intron excluded)

R: number of raw reads

### RT-PCR and Sanger sequencing

First strand cDNA synthesis was performed with 0.5 μg total RNA using a High Capacity cDNA Reverse Transcription kit (Life Technologies) according to the manufacturer’s instructions. PCR was performed in a 25-μL reaction mix containing 1× AmpliTaq Gold® 360 Master Mix (Life Technologies), 200 μM of each primer, and 2 μL of cDNA. The PCR cycling conditions were: 10-min incubation at 95°C, followed by 40 cycles of 94°C for 30 s, 60°C for 30 s, 72°C for 60 s, and a final incubation at 72°C for 10 min. The resulting PCR products were digested with ExoSAP-IT reagent (Affymetrix, Cleveland, OH) and then sequenced in forward and reverse directions with a BigDye Terminator Kit (Life Technologies) and an ABI 3730XL DNA analyzer (Life Technologies), following the manufacturer’s instructions. The sequencing data were analyzed for mutations after assembly and quality calling with SeqScape sequence analysis software (version 2.5; Life Technologies). The RT-PCR primers used for fusion gene confirmation were: *1) ZNF207/HER2*, Forward: 5'-CTGAAGCCGTGGTGCTGGTATTGTA-3', Reverse: 5'-TGGGCATGTAGGAGAGGTCAGGTTT-3'; 2) *MDK/HER2*, Forward: 5'-GTTTGAGAACTGGGGTGCGTGTGAT-3', Reverse: 5'-AGACCATAGCACACT CGGGCACA-3'; 3) *NOS2/HER2*, Forward: 5’-CAAGCCCCACAGTGAAGAACATCTG-3', Reverse: 5'-TGCTGGAGGTAGAGTGGTGAACAGG-3'.

### Whole genome sequencing

DNA was extracted from the frozen tissues using a Puregene DNA extraction kit (Qiagen) and quantified using a PicoGreen fluorescence assay (Qubit; Invitrogen). To conduct whole genome sequencing, 2 μg of DNA were required for each sample. After electrophoresis, DNA fragments of the desired length were gel purified. Adapter ligation and DNA cluster preparation were performed and subjected to Illumina Hiseq2000 sequencing. Two paired-end libraries with an insert size of 500 bp were prepared for all samples, after which four lanes from each library were subjected to whole genome sequencing. Raw image files were processed by Illumina Pipeline for base calling with default parameters, and the sequences of each individual were generated as 90-bp paired-end reads. Raw sequence data was mapped to the reference human genome (hg19) using Bowtie 2. The total mapping rate was >90%, and the average coverage was about 30×. Unmapped reads were then used to conduct genomic fusion detection with FusionMap [16].

### Analysis of *HER2*, *BRAF*, *KRAS*, or *PI3K* mutations

*HER2*, *BRAF*, *KRAS*, and *PIK3CA* gene mutations from the RNAseq data were analyzed using ArrayStudio software (http://www.omicsoft.com/array-studio.php). Allele frequencies below 10% were removed in case of potential false positive. The mutation status was further confirmed by the whole genome sequencing data.

### Immunohistochemistry (IHC)

The primary antibodies used to detect the cytoplasmic domain of *HER2* were purchased from Merck and Abcam, and the antibody used to detect the external domain of HER2 was purchased from Abnova. All of the collected tissues were fixed in FFPE blocks. Xenograft and cell-block sections were cut at 3 μm and human sections were cut at 4 μm for the HER2 IHC study. Paraffin sections were dewaxed and rehydrated in a Leica XL autostainer. Following antigen retrieval, the sections were incubated with 10 min of endogenous peroxidase block (DAKO), 60 min of primary antibodies, 30 min of EnVision System-HRP labeled polymer anti-mouse (DAKO), and 10 min of diaminobenzidine substrate (DAKO K3468), in that order. Finally, the sections were counter-stained, dehydrated, cleared, and mounted with coverslips in a Leica XL autostainer workstation. A HercepTest™ (DAKO) was used to detect the membranous expression of HER2, following standard procedures. Each slide was evaluated and scored on a 0–3 scale, following uniform guidelines developed for GC HER2 scoring from ToGA trials [13].

### Fluorescent in *situ* hybridization (FISH)

A multicolor FISH assay for *HER2* split screening was conducted via a dual-probe FISH break-apart test. The N-terminal and C-terminal probes for *HER2* were generated internally by directly labeling BAC (N-terminal: RP11-98 J2; C-terminal: RP11-1044P23) DNA respectively with Green-dUTP (ENZO, Cat # 02 N32-050) and Red-dUTP (ENZO, Cat #02 N34-050). A CEP17 spectrum aqua probe (Vysis, Cat #32-131017) for the centromeric region of chromosome 17 was used as an internal control of the *HER2* break-apart probes. Multicolor FISH was also used to confirm *HER2* gene fusion with certain partner genes. The *ZNF207* and *NOS2* FISH probes were generated internally by directly labeling BAC (*ZNF207*: RP11-55 J8; *NOS2*: RP11-696H14) DNA with gold 525-dUTP (ENZO, Cat #ENZ42843). The FISH assays were performed as previous reported [17]. Briefly, the assay was run on 4-μm dewaxed and dehydrated FFPE TMAs. A SPoT-Light tissue pretreatment Kit (Invitrogen, Cat #00-8401) was used for the pretreatment (boiled in reagent 1 for ~18 minutes, then coated with reagent 2 for ~14 minutes, minor time adjustments were made for individual samples). The sections and probes were co-denaturated at 79°C for 6 minutes and then hybridized at 37°C for 48 hours. After a quick post wash off process (0.3% NP40/2xSSC at 75.5°C for 2 minutes, twice in 2 × SSC at room temperature for 2 minutes), the sections were mounted with 0.3 μg/ml DAPI (Vector, Cat #H-1200) and stored at 4°C, avoiding light for at least 30 minutes prior to observation. The FISH signals were observed using a fluorescence microscope equipped with the appropriate filters, allowing visualization of the intense red/green/gold signals of the target genes, the intense aqua centromere signals, and the blue counterstained nuclei. A minimum 100 nuclei were scored for each sample. Only nuclei with a minimum of two green and two red signals were scored. In the break-apart assay, fused N-terminal (green) and C-terminal (red) signals represent a normal *HER2* gene. *HER2* amplification status was defined as a ratio of fused *HER2* signals to CEP17 (aqua) ≥2. The following situations indicated the presence of *HER2*-involved fusion: 1) broken apart: more than one set of broken-apart N-terminal and C-terminal signals in ≥10% tumor cells; 2) N-terminal deletion: more C-terminal signals in addition to fused and/or broken-apart signals in ≥30% tumor cells. Then, a multicolor FISH assay was performed on *HER2* broken apart or N-terminal-deleted positive cases. The *HER2* fusion was confirmed when *HER2* C-terminal red signals co-localized with a certain partner gene’s gold signals.

### Vector construction, cell culture, transduction, and transformation studies

*ZNF207-HER2* and *MDK-HER2* fusion cDNA were synthesized (Additional file 1: Files S1 and S2) at Generay (Shanghai, China). The products were subcloned into PLVX lentiviral vector (Sunbio, China). The integrity of the inserted cDNA was verified by Sanger sequencing of the constructs. Lentiviruses expressing *ZNF207-HER2* and *MDK-HER2* fusions were produced according to the manufacturer’s instructions. NIH/3 T3 fibroblast cells were infected with lentiviruses expressing empty vector, and *ZNF207-HER2* and *MDK-HER2* fusions were treated with puromycin (2 μg/ml) for two weeks. NIH/3 T3-resistant cells were seeded on 96-well plates (2000 cells/well) in 0.33% agar in complete medium. The expression of exogenous HER2, ZNF207-HER2, and MDK-HER2 proteins and phosphorylation of HER2 were examined by immune blot analysis. The overnight cell cultures in liquid or soft agar medium were treated with lapatinib, T-DM1 (synthesized at ChemPartner, Shanghai, China) for 3 and 14 days, respectively. The cell growth rate was measured by MTS assay according to the manufacturer’s instructions (Promega).

### Immunoblot analysis

Total cellular extracts from the cell lines were prepared in an SDS lysis buffer supplemented with protease inhibitors and phosphatase inhibitors (Sigma). Protein samples were fractionated by SDS-PAGE and blotted onto polyvinylidene difluoride membranes (Millipore). After incubation with the indicated antibodies at 4°C overnight, the blots were detected with the relevant horseradish peroxidase-conjugated anti-mouse or anti-rabbit IgG antibody and enhanced chemilluminescence (GE Healthcare). The antibody information used in the Western blot assays are included were as follows: pHER2 (Y1221/1222) (CST, Cat #2243, diluted 1:1000); HER2 (CST, Cat #2165, diluted 1:1000); pErk1/2 (T202/Y204) (CST, Cat #4376, diluted 1:1000); Erk1/2 (CST, Cat #9102, diluted 1:1000); pAKT (Ser473) (CST, Cat# 9271, diluted 1:1000); and AKT (CST, Cat #9272, diluted 1:1000).

### Trastuzumab emtansine (T-DM1) receptor binding assay

Approximately 5 × 10^5^ NIH/3T3 cells expressing vector control, wild-type HER2, ZNF207-HER2, or MDK-HER2 were collected using enzyme-free cell dissociation buffer (Invitrogen). After blocking with 10% donkey serum for 30 minutes at 4°C, the cells were incubated with 10 μg/mL T-DM1 (ChemPartner) at 4°C for one hour. The cells were rinsed three times with wash buffer (0.5% BSA in PBS) and further incubated with 10 ug/mL Alex488 labeled donkey anti-human IgG (Jackson immunology) at 4°C for one hour. After rinsing five times with wash buffer, the mean intensity of the fluorescence was detected by FACSCanto (BD). The receptor binding was qualitatively evaluated by the peak shift in the histogram.

### *In vivo* efficacy study in xenograft models

6- to 8-week-old female nude (*nu/nu*) mice (Vital River, Beijing, China) were used for *in vivo* efficacy studies. All experiments using immunodeficient mice were carried out in accordance with the guidelines approved by the Institutional Animal Care and Use Committees. NIH/3 T3 cells expressing *ZNF207-HER2, MDK-HER2*, or a control vector were inoculated subcutaneously into female nude mice. Tumor-bearing mice with tumors ranging 100–200 mm^3^ in size were selected randomly and placed in groups according to their tumor volume and body weight (eight animals per group) for treatment. Trastuzumab (15 mg/kg) was administrated by intravenous injection twice a week. The xenograft tumors were measured in two perpendicular diameters with a caliper, and tumor volumes (TV) were calculated using the formula TV = (length × width^2^)/2. Percentage of tumor growth inhibition (%TGI) was calculated using the formula [1-(change of tumor volume in treatment group/change of tumor volume in control group)] × 100, and was used to evaluate anti-tumor efficacy. Student’s t tests were used to compare the TGI of the treatment group with that of the control group. Statistical tests were two sided, with P < 0.05 considered significant.

## Results

### Novel *HER2* fusion genes identified in HER2-positive gastric cancer

**Figure 1:**
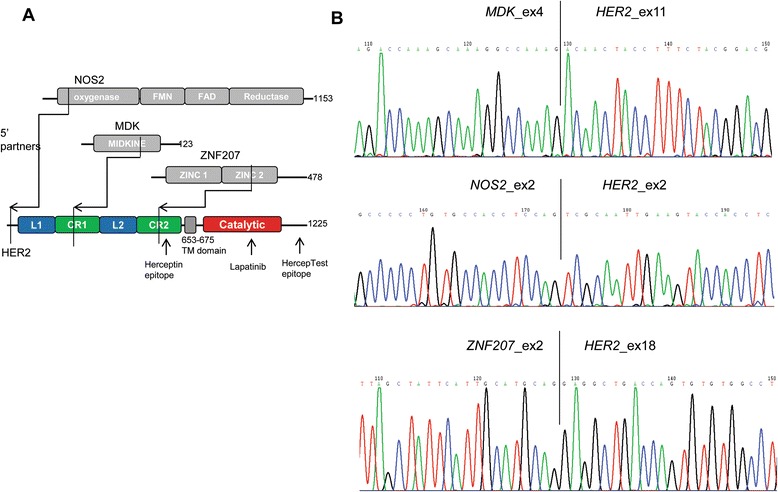
Identification of HER2 fusions in gastric cancer. **A.** Schematic illustration of the wild-type HER2 protein and the three fusions identified in this study. The breakpoints for each fusion are indicated by arrows. **B**. Sanger sequencing of the fusion junctions.

To uncover the genetic aberrations that might confer resistance to trastuzumab treatment in GC, we performed whole-transcriptome sequencing (RNAseq) on 21 HER2-positive tumor specimens from Chinese GC patients whose tumors were surgically removed and who were treatment naïve, using the HiSeq2000 system (Illumina). A number of candidate fusion transcripts were identified with more than three chimerical reads, which were subsequently followed by RT-PCR/Sanger sequencing confirmation. This led to the identification of three *HER2* (chr17q12) in-frame fusion transcripts with 5’ partners of *ZNF207* (chr17q11.2), *MDK* (chr11p11.2), or *NOS2* (chr17q11.2) in three HER2-positive GC samples of GC196, 431-9540474 T, and GC334, respectively (Figure 1A, B and Table 1).

**Table 1:** **A summary of patients with gastric cancer harboring** ***HER2*** **fusions**

**Samples**	**Country**	**Sex**	**Age**	Fusions*	**TNM**	**Pathological type**
GC196	China	M	76	*ZNF207*_ex2/*HER2*_ex18	T3N1M0	Mixed
431-9540474 T	China	M	63	*MDK*_ex4/*HER2*_ex11	T2N2M0	-
GC334	China	F	62	*NOS2*_ex2/*HER2*_ex2	T3N2M0	Diffused

*The amplification of *MDK-HER2* was determined by aCGH, whereas the other two *HER2* fusion amplifications were defined by FISH and IHC assays.

**Figure 2:**
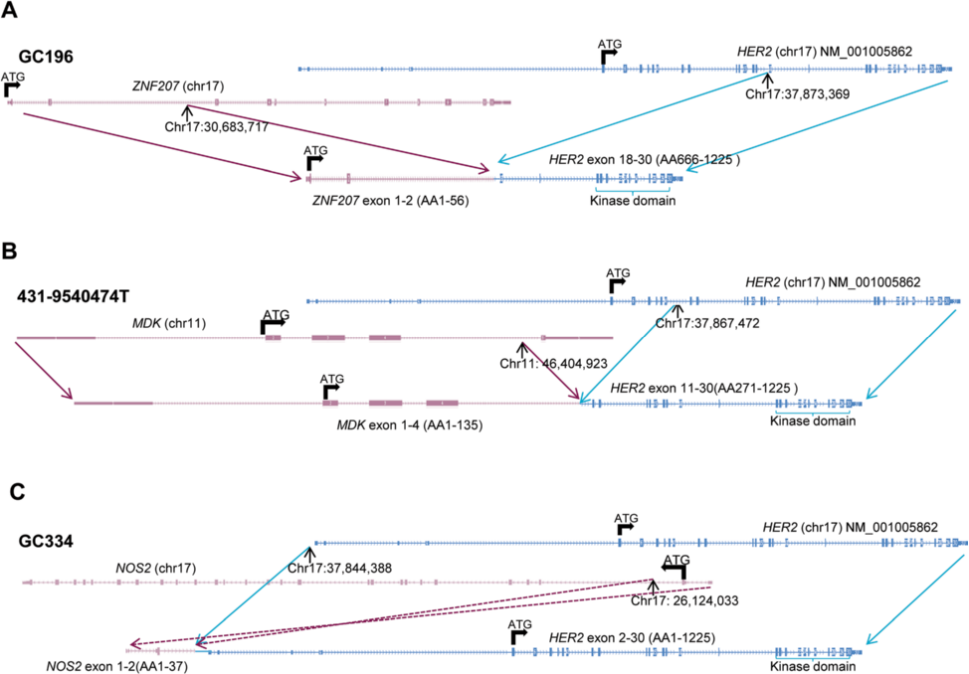
Schematic of genomic fusion structure of the HER2 fusion. **A.** Genomic fusion structure for sample GC196 harboring *ZNF207-HER2* fusion **B**. Genomic fusion structure for sample 431-9540474 T harboring *MDK-HER2* fusion **C**. Genomic fusion structure for sample GC334 harboring *NOS2-HER2* fusion. The genomic structure was discovered by whole genome sequencing.

To understand the genomic alterations of these fusion transcripts, tumor DNA samples from the three corresponding GC patients were analyzed by whole genome sequencing. Consistent with the data from the RNAseq analysis, three *HER2* fusion genes—*ZNF207* (exon 1–2)/*HER2* (exon 18–30), *MDK* (exon 1–4)/*HER2* (exon 11–30), and *NOS2* (exon 1–2)/*HER2* (exon 2–30)—were further confirmed in these tumor DNA samples (Figure 2). These results demonstrate the existence of three novel *HER2* gene fusions in this cohort of HER2-positiv GC patients.

### Amplification and overexpression of the *HER2* fusions in GC

In the GC196 sample, over 100 reads were detected for *ZNF207* genomic sequences only composing the 5’ partner and *HER2* sequences only composing the 3’ partner of the *ZNF207-HER2* fusion; low reads (~20) were captured for the genomic sequences outside the fusion gene. In the GC334 specimen, in addition to the high reads (>1800) of the fusion partners of *NOS2-HER2* fusion that were detected, an average of 500 reads was also captured for the entire genomic sequence of wild-type *HER2* gene.

**Figure 3:**
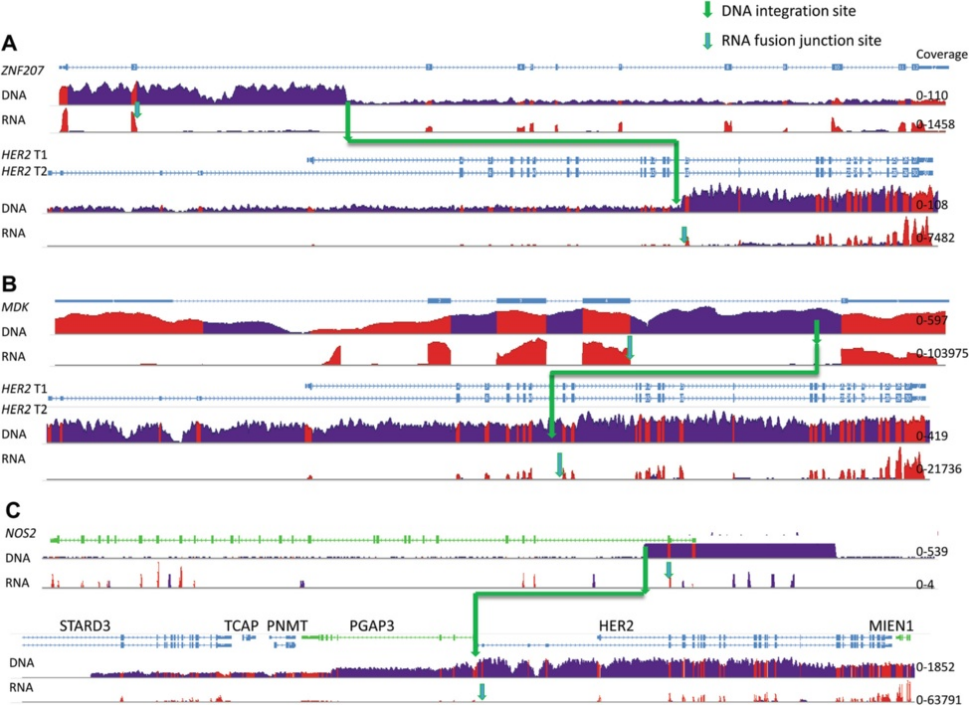
Illustration of DNA coverage and mRNA expression levels of HER2 and fusion partners. **A.** DNA coverage and mRNA expression levels of *HER2* and *ZNF207* in sample GC196. **B**. DNA coverage and mRNA expression levels of *HER2* and *MDK* in sample 431-9540474 T **C**. DNA coverage and mRNA expression levels of *HER2* region (including *HER2* and nearby gene *STARD3*, *TCAP*, *PNMT*, *PGAP3* and *MIEN1*) and fusion partner *NOS2* in sample GC334. The DNA integration sites were marked in green arrows and the RNA fusion junction sites were marked in cyan arrows. Exon coverage was shown in red and intron coverage was shown in blue. The coverage range for each gene was given in the right side of the figure. Average coverage for WGS is 30X and average coverage for RNASeq is 150X.

Next, we assessed the relative mRNA expression levels of the three amplified fusion genes. Consistent with the whole genome sequencing data, high levels of mRNA expression of the three fusion variants were obtained in the three HER2*-*fusion-positive tumor specimens (GC196 FPKM 346, 431-9540474 T FPKM 1359, and GC334 FPKM 2805). In the tumor harboring *ZNF207-HER2*, the increased expression of *HER2* mRNA was observed only after the fusion site, whereas the increased expression of *ZNF207* mRNA was detected only before the fusion site, further confirming that the amplification of the *ZNF207-HER2* fusion was a homogeneous event in the sample (Figure 3A). In contrast, in the tumor harboring *MDK-HER2*, overexpressed transcripts were detected for *MDK-HER2* fusion, wild-type *HER2*, and wild-type *MDK*, consistent with the heterogeneity populations of amplified wild-type *HER2* with *MDK-HER2* (Figure 3B). A similar observation was made in the GC334 sample, in which overexpression of both *NOS2-HER2* and wild-type *HER2* were detected (Figure 3C).

**Figure 4:**
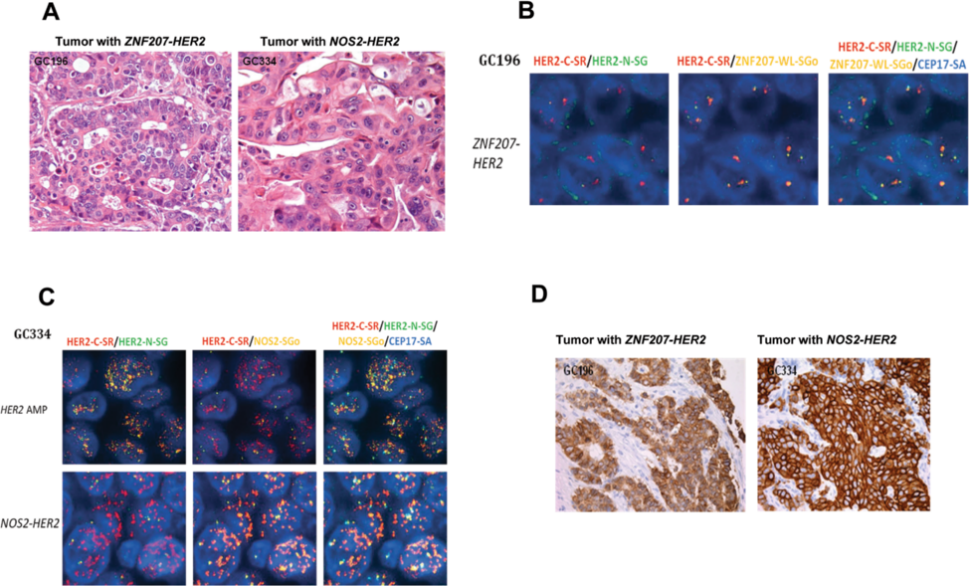
Amplification and over-expression of HER2 fusions in gastric tumors. **A.** Histological characterization of human gastric carcinomas. Representative images from H&E staining of GC196 and GC334 primary tumors. **B**. *ZNF207-HER2* gene fusion in primary tumor GC196. Representative images of break-apart FISH on GC196 shows normal copy of *HER2* N-terminal (green signals), coexistence of amplified *HER2* C-terminal (red signals) and amplified *ZNF207* (gold signals). Red and green signals represent C-terminal (HER2-C-SR) and N-terminal (HER2-N-SG) of *HER2* gene, respectively; gold signals represent whole length of *ZNF207* gene (ZNF207-WL-SGo); aqua signal represent *CEP17* as internal control. Cell nuclei were counterstained with DAPI. **C**. *NOS2-HER2* gene fusion in tumor GC334 primary tumor. Representative images of break-apart FISH on GC334 show *NOS2-HER2* fusion with heterogeneity. The upper lane showed tumor cells with both *HER2* N-terminal (green signals) and C-terminal (red signals) amplified and normal copy number of *NOS2* (gold signals). The lower lane showed tumor cells with normal copy of *HER2* N-terminal (green signals), coexistence of amplified *HER2* C-terminal (red signals) and amplified *NOS2* (gold signals). Red and green signals represent C-terminal and N-terminal of *HER2* gene; gold signals represent *NOS2* gene; aqua signal represent *CEP17* as internal control. Cell nuclei were counterstained with DAPI. **D**. IHC analysis of HER2. Representative images showed HER-2 IHC strong membrane staining (+++) on GC196 and GC334 primary tumors.

Given that the tumor samples harboring the three fusions were all HER2-positive, we first developed a multicolor fluorescence *in situ* hybridization (FISH) assay to assess the genetic amplification status of the *ZNF207-HER2* and *NOS2-HER2* fusion genes, as well as to dissect their relationship to the wild-type *HER2* gene in the GC196 and GC334 samples, respectively. Based on hematoxylin and eosin staining, both primary tumors with the two *HER2* fusions were defined as adenocarcinoma by pathologists (Figure 4A). The multicolor FISH assay detected the N-terminal and C-terminal of the *HER2* gene, along with the *ZNF207* or *NOS2* gene. As shown in Figure 4B, the *ZNF207-HER2* fusion gene was amplified homogeneously without wild-type *HER2* amplification in the GC196 sample. However, co-amplification of both *NOS2-HER2* and wild-type *HER2* were observed in the GC334 specimen, but in separate tumor cell populations (Figure 4C). The co-localization of 5’ partners and the C-terminus of *HER2* gene by the FISH analysis not only further confirmed the genomic fusions of the involved genes, but also indicated the intratumoral heterogeneity within the *HER2*-amplified tumor. For the *MDK-HER2* fusion, although a formalin-fixed and paraffin-embedded (FFPE) sample of 431-9540474 T was unavailable for the multicolor FISH analysis, the whole genome sequencing data suggested a mixture of tumor cell populations harboring gene amplifications of wild-type *HER2*, *MDK-HER2*, and wild-type *MDK* (Figure 3).

Lastly, we performed immunohistochemistry (IHC) with a HercepTest™ in FFPE samples of GC196 and GC334. Strong staining (IHC 3+) was detected in both samples (Figure 4D), indicating an overexpression of the HER2 proteins. A summary of the HER2 fusion status was shown in Table 2.

**Table 2:** **A summary of the** ***HER2*** **fusion status**

**Sample ID**	**Fusion Type(RNA)**	**DNA structure**	**5' fusion partner status**			**HER2 status**	
GC196	ZNF207_exon2 /HER2_exon18	*ZNF207*_intron2/*HER2*_intron17	*ZNF207*	mRNA expression	High	mRNA Expression	High
				Gene copy	N Ter AMP	Gene Copy	C Ter AMP
				Protein level	-	Protein level	IHC 3+
431-9540474 T	MDK_exon4 -/HER2_exon11	*MDK*_intron4/*HER2*_intron10	*MDK*	mRNA expression	High	mRNA Expression	High
				Gene copy	tAMP	Gene Copy	tAMP
				Protein level	-	Protein level	-
GC334	NOS2_exon2 /HER2_exon2	*NOS2*_intron2/*HER2*_5’UTR	*NOS2*	mRNA expression	Low	mRNA Expression	High
				Gene copy	N Ter AMP	Gene Copy	tAMP
				Protein level	-	Protein level	IHC 3+

N Ter AMP: N terminal amplification.

C Ter AMP: C terminal amplification.

tAMP: total amplification.

Together, these data demonstrate the gene amplification of the *HER2* fusions, which correlated with dis-regulation of both mRNA and protein expressions of the fusion variants. In addition, the HercepTest™ did not distinguish the fusion proteins from the wild-type *HER2*.

### Oncogenic driver activity of the *HER2* fusions

A number of genetic aberrations with oncogenic properties have been reported recently in GC cases [8]. Thus, we aimed to determine whether the *HER2* fusions overlapped with the known oncogenic alterations. RNAseq and WGS data showed that the three *HER2-*fusion-positive GC patients were negative for *HER3*, *BRAF*, *KRAS*, *PI3KCA*, and *HER2* mutations, and negative for amplifications of *FGFR2* and c*MET (data no shown)*, which are common genetic alterations identified in GC [18]. The mutually exclusive nature of *HER2* fusions from these known oncogenic alterations suggests that the *HER2* fusions are oncogenic drivers. In addition, sequence analysis revealed that proteins encoded by *ZNF207-HER2* and *MDK-HER2* fusion variants contained a partial extracellular domain, a transmembrane domain, and a full kinase domain of HER2 (Additional file 1: Files S1 and S2), whilst the predicated fusion protein of *NOS2-HER2* would be a full-length HER2 protein as result of a stop codon that is introduced prior to the *HER2* start codon (Additional file 1: File S3). Based on the sequence predication that *NOS2-HER2* fusion encodes a full-length HER2 without NOS2, our efforts on function characterization of these HER2 fusion proteins were then focused on *ZNF207-HER2* and *MDK-HER2* fusions.

**Figure 5:**
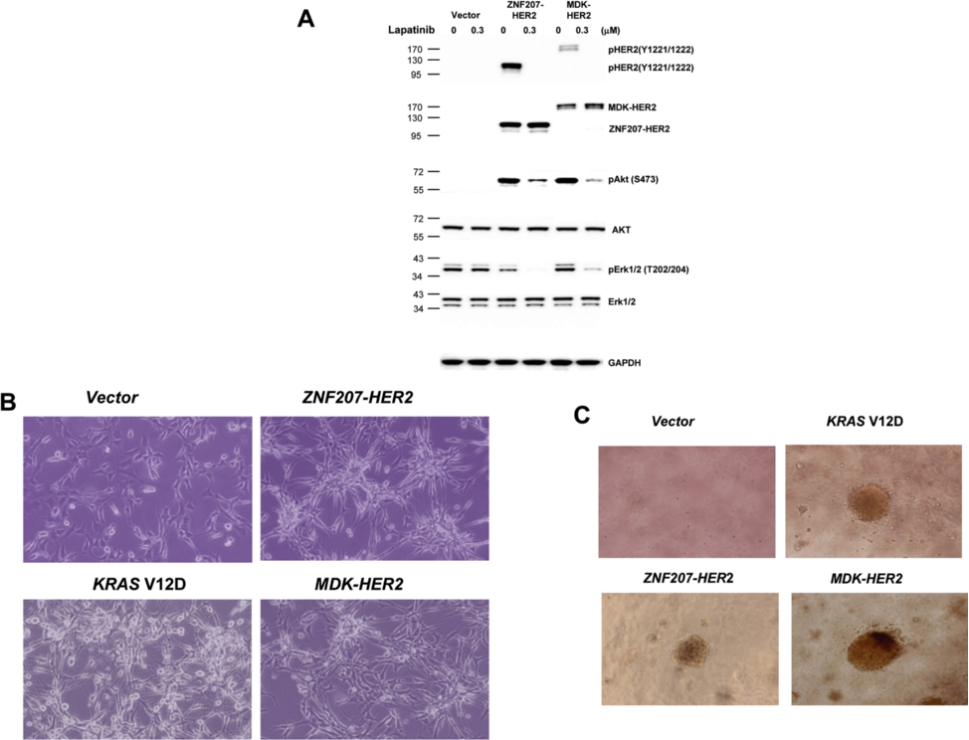
**Transformation of NIH/3T3 cells by** ***ZNF207-HER2*** **and** ***MDK-HER2*** **fusions. A**. Modulation of HER2 signaling in NIH/3 T3 cells expressing *HER2* fusions. Cell lysates collected from cells stably expressing *HER2* and *HER2* fusions were subjected to western blotting analysis with antibodies against phospho and total HER2, Akt and Erk. **B**. Exogenous expression of *ZNF207-HER2* and *MDK-HER2* led to transformational morphological changes. Cells were photographed under a phase-contrast light microscope (×150) under identical conditions. **C**. Anchorage-independent growth of NIH/3 T3 cells expressing *HER2* fusions. Cells were seeded in soft agar culture in 96-well plates for 14 days and colony formation was photographed under a phase-contrast light microscope (×150). The results are representative from three independent experiments.

Given the presence of the HER2 dimerization domain [19,20], ZNF207-HER2 and MDK-HER2 fusion variants are likely to form homodimers in a manner similar to that of amplified wild-type HER2. Consistently, when MDK-HER2 and ZNF207-HER2 variants were ectopically expressed in NIH/3 T3 cells, the autophosphorylation sites Tyr1221/1222 at the C-terminus of HER2 involved in the activation of HER2 signaling were phosphorylated in a similar manner as wild-type HER2 [21] (Figure 5A). This result indicates an aberrant activation of HER2 kinase by the fusions with *MDK* or *ZNF207*. Downstream-signaling AKT was also phosphorylated by the fusion variants (Figure 5A). Phospho-HER2^Y1221/1222^ and phospho-AKT^S473^ were suppressed by Lapatinib (Figure 5A), thus predicting the sensitivity of GC harboring the *HER2* fusions to the HER2 kinase inhibitor, such as Lapatinib.

In addition to the induction of phosphorylation of Tyr1221/1222, the exogenous expression of *ZNF207-HER2* or *MDK-HER2* fusions in NIH/3 T3 cells also induced transformational morphology (Figure 5B); anchorage-independent growth of the cells *in vitro*, which was comparable to the oncogenic phenotypes caused by mutant *KRAS* (V12D) (Figure 5C). Together, these results demonstrate the oncogenic properties of the *ZNF207-HER2* and *MDK-HER2* fusions in GC.

### Different binding and responsiveness of the HER2 fusions to T-DM1 and trastuzumab

**Figure 6:**
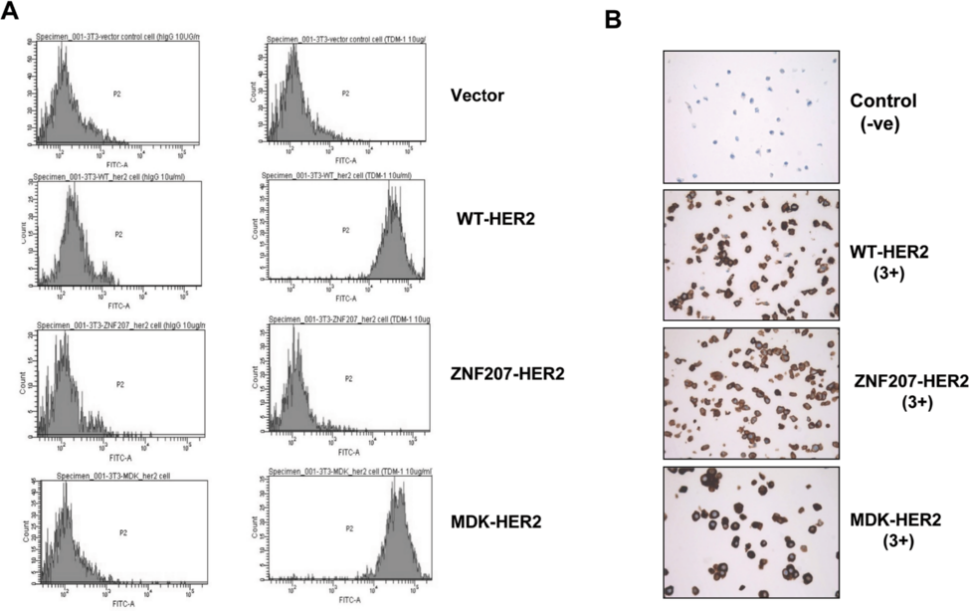
**Binding of the HER2 fusions with T-DM1. A**. Cells were incubated with T-DM1 at 4°C for 1 hr and the bound T-DM1 was measured by FACS with an Alex488 labeled secondary antibody. The results are representative from three independent experiments. **B**. IHC analysis of HER2 with HercepTest™).

To determine whether the 5’ partners fused to a truncated HER2 extracellular domain affects their binding ability to trastuzumab in the two HER2 fusion variants, we used T-DM1 to assess its binding to NIH/3 T3 cells ectopically expressing wild-type HER2, MDK-HER2, or ZNF207-HER2. Our results clearly showed that under those conditions, the ZNF207-HER2 fusion lost its ability to bind to T-DM1, while MDK-HER2 bound to T-DM1 in a manner similar to that of wild-type HER2 (Figure 6A). Because the receptor binding assay was performed using the engineered cells, it might be argued that the impaired binding of ZNF207-HER2 could be due to improper cellular localization of the fusion protein. To address this issue, cellular localization of ectopically expressed MDK-HER2 or ZNF207-HER2 in the NIH/3 T3 cells was assessed by HercepTest™. As shown in Figure 6B, strong membrane staining (IHC 3+) was observed in the NIH/3 T3 cells expressing *MDK-HER2* or *ZNF207-HER2*, similar to the results observed in the original primary tumor samples (Figure 4D), thus further supporting the inability of ZNF207-HER2 to bind to T-DM1.

**Figure 7:**
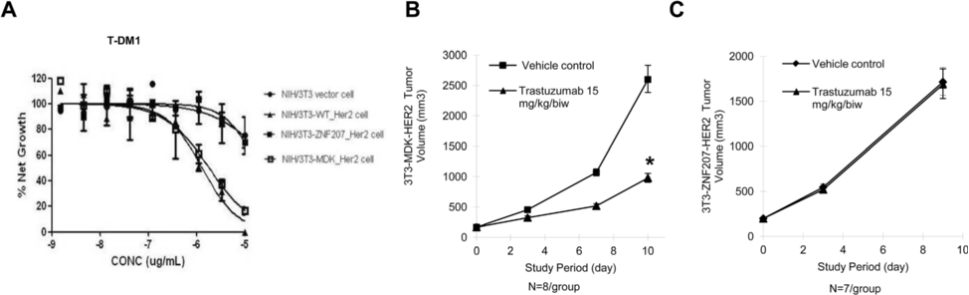
**Response of NIH/3T3 cells expressing** ***HER2*** **fusions to T-DM1 and trastuzumab. A**. Response of NIH/3 T3 cells expressing *HER2* fusions to T-DM1. Cells were treated with T-DM1 at indicated concentrations for 72 hrs and cell proliferation was measured by MTS assay. The error bars represent SD. B-C. Anti-tumor efficacy of trastuzumab in xenograft models expressing the HER2 fusions. Nude mice bearing NIH/3 T3 xenografts stably expressing the *MDK-HER2* **(B)** or *ZNF207-HER2* fusion variant **(C)**, were treated with trastuzumab at 15 mg/kg biweekly and the tumor size was measured with a caliper. The error bars represent SEM and students’ T-tests were used to compare the growth rate in the treatment group with that in the control group *: *P* < 0.05.

The different binding capabilities of the HER2 fusions to T-DM1 predict different responsiveness of GC cells with the *HER2* fusions to trastuzumab-based anti-HER2 therapies. T-DM1 was effective in inhibiting the growth of cells expressing wild-type HER2 or MDK-HER2 fusion, but this inhibitory effect was significantly impaired in the cells expressing the ZNF207-HER2 fusion (Figure 7A), indicating an intrinsic resistance mechanism to trastuzumab-based therapy. The resistance of ZNF207-HER2 to T-DM1 was further confirmed in *in vivo* efficacy study. We tested the anti-tumor efficacy of trastuzumab in NIH/3 T3 xenografts expressing the ZNF207-HER2 or MDK-HER2 fusion variant. As expected, the trastuzumab treatment resulted in significant tumor growth inhibition in the xenografts expressing the MDK-HER2 fusion (TGI = 67%) (Figures 7B), but it showed no efficacy in the xenografts expressing the ZNF207-HER2 fusion (TGI = 2%) (Figures 7C), thus supporting the resistant mechanism to trastuzumab in GC.

In summary, our results clearly demonstrate that the ZNF-207-HER2 fusion does not respond to trastuzumab, due to the loss of its binding ability.

## Discussion

Several studies have recently used NGS to understand the molecular basis of GC, and a number of previously unknown genetic alterations have been reported [22], including genetic fusions of *HER2* in human GC cell lines. For example, two *HER2* fusions were identified at the same time in human GC cell line MKN7 (HER2 positive): a fusion between *CDK12* exon 12 and *HER2* intron 4, and a second fusion between *NEUROD2* exon 1 and *HER2* exon 8 [22]. However, there was no direct evidence in that report that demonstrated the oncogenic driver of these *HER2* fusions. In the current study, we performed a whole-transcriptome sequencing of 21 HER2-positive GC tumor samples taken from Chinese patients, and discovered three *HER2* fusion transcripts due to *HER2* gene fusions. Two of them, ZNF207-HER2 and MDK-HER2 were truncated in the N-terminal extracellular domains, but they remained intact in the kinase and transmembrane domains of HER2. The amplification and overexpression of the three *HER2* gene fusions were detected in the primary GC samples by multicolor FISH and RNAseq or IHC-based HercepTest™ analysis. The ectopic expression of *ZNF207-HER2* and *MDK-HER2* in the NIH/3T3 cells led to a constitutive activation of *HER2* and downstream signaling, and thus, cell transformation *in vitro* and tumorigenesis *in vivo*, demonstrating the oncogenic driver of the fusions. Furthermore, the xenografts ectopically expressing *MDK-HER2* but not *ZNF207-HER2* were sensitive to trastuzumab*.* Interestingly, the *HER2* fusions were found to be mutually exclusive with mutations of *PI3KCA*, *BRAF*, *KRAS*, and *HER3* and amplifications of *FGFR2* and *c-MET*. Collectively, our data confirmed for the first time the presence of oncogenic *HER2* arrangements in patients with GC. The *ZNF207-HER2* fusion represents a novel intrinsic resistance mechanism to trastuzumab-based anti-HER2 therapy, due to the loss of binding ability to trastuzumab.

Despite the overall survival benefit achieved with trastuzumab in GC patients carrying *HER2* amplification, a significant portion of the patients do not respond clinically to the treatment, and there is little understanding of the molecular mechanism underlying this intrinsic resistance. In contrast, the understanding of the mechanisms of both intrinsic and acquired resistance to *HER2* inhibitors in HER2-positive BC is far more advanced [15,23]. For example, some intrinsic resistance mechanisms affect the ability of HER2 inhibitors to directly engage HER2 in BC; a truncated form of HER2, p95, lacking the trastuzumab binding region [24,25]; a splice variant that eliminates exon 16 (*HER2*-Δ16) in the extracellular domain of the HER2 receptor, preventing disruption of HER2 homodimers upon binding by trastuzumab [26]; In our study, we did not assess p95, but we did find *HER2*-Δ16 in a GC tumor sample, which naturally harbored *HER2*-Δ16. Surprisingly, its corresponding patient-derived gastric cancer xenograft (PDGCX), which retains *HER2*-Δ16 (data not shown), responded well to trastuzumab treatment, with a significant tumor regression being observed (data not shown). Given the lack of clinical evidence of association between *HER2*-Δ16 and resistance to trastuzumab in BC, as well as the lack of preclinical data on the anti-tumor efficacy of trastuzumab in xenografts carrying *HER2*-Δ16 [26], our data suggest that *HER2*-Δ16 was not a resistance mechanism to trastuzumab in GC when tested in the PDGCX model. Additional *HER2*-Δ16 positive PDGCX models are warranted to confirm this observation further.

Surprisingly, in addition to the *HER2* fusions, we also found two recurrent in-frame *BRAF* fusion transcripts, *BAIAP2L1-BRAF* (data not shown), in another cohort of *HER2*-amplified GC patients. Although further work is needed to demonstrate its oncogenic activity and sensitivity to trastuzumab or BRAF inhibitor, the findings suggest that BAIAP2L1-BRAF could be a potential resistance mechanism to trastuzumab, as it maintains an intact BRAF kinase domain [27].

The current clinical protocol for selecting HER2-positive patients is based on FISH positivity or an IHC (HercepTest™) score of 3+ (HercepTest™ score of 2+ needs further FISH confirmation). *HER2* gene amplification is determined by the ratio between the numbers of signals from the hybridization of the *HER2* gene probe (covers the whole *HER2* gene) and the number of signals from the hybridization of the reference chromosome 17 centromere probe [28]. The antibody used in the HercepTest™ recognizes the HER2 epitope located at the HER2 intracellular site, which is also covered by all three *HER2* fusions (Figure 1A). Therefore, the current HER2 tests cannot distinguish between the amplifications of the HER2 fusions and that of the wild-type. The use of multicolor FISH and RT-PCR assays to detect the *HER2* fusions developed in this study demonstrated the feasibility of detecting *HER2* fusions as clinical biomarkers in either FFPE or frozen surgical GC specimens. This finding warrants further clinical validation of the novel resistant mechanism of the ZNF207-HER2 fusion to trastuzumab-based therapy.

In addition to the antibody, a number of small-molecule HER2 kinase inhibitors are available: lapatinib, an approved agent for HER2-positive BC patients, and afatinib and neratinib, two irreversible kinase inhibitors, currently in phase III clinical trials for HER2-positive BC. The modulation of AKT signaling by lapatinib in the cells expressing the HER2 fusions (Figure 5A) suggests that the small-molecule inhibitor against HER2 kinase is a potential option for cancer patients with the *HER2* fusions. This notion is further supported by our observations that NIH/3T3 cells expressing either ZNF207-HER2 or MDK-HER2 showed to be sensitivity to lapatinib, afatinib, and neratinib *in vitro* (data not shown)*.*

Besides the functional and phonotypical heterogeneity, emerging evidences indicate that genetic heterogeneity among tumor cells contributes to the advantages for survival, proliferation, metastasis and resistance to anti-cancer therapies. Recently, Tajiri et al. examined 475 GC samples using multiple ligation-dependent probe amplification (MLPA) and FISH analysis and revealed intratumoral heterogeneity of *HER2* amplification in 41% (21/51) of *HER2*-amplified tumors. The mutually exclusive co-amplification of *HER2* with *EGFR*, *FGFR2*, *FGFR2* and *MET* was also observed respectively in some of the tumors, suggesting the potential challenges for design of targeted-therapy approaches [29]. The homogenous expression of ZNF270-HER2 supports the driver role of this fusion gene, which is consistent with our experimental results. However, the advantage of co-amplification of *MDK-HER2* and *NOS2-HER2* with wild *HER2* (intra-tumor heterogeneity) is yet to be uncovered. Further studies to explore the responsiveness of the HER2 fusions to combination of trastuzumab with pertuzumab or chemotherapies may lead to additional insights into the impact of the HER2 fusions to anti-HER2 therapies.

It is noteworthy that the HER2 positive GC samples used for this study were collected prior the introduction of trastuzumab to China, thus we lack evidence for clinical response of the tumors harboring HER2 fusions to trastuzuamb. Further studies on GC samples from patients treated with trastuzumab at different stages will help to confirm the effect of the HER2 fusions to trastuzmab therapy and their oncogenic property.

Although further large-scale clinical investigations are needed to understand the clinical prevalence in GC patients, our data on *ZNF207-HER2*, along with the discovery of two recurrent *BAIAP2L1-BRAF*, strongly indicate a large degree of molecular heterogeneity, even in the well-defined HER2-positive segment, representing potential *de novo* resistance to trastuzumab-based GC therapies. In addition, whether a similar mechanism would exist in BC also needs to be exploited.

## Conclusions

In summary, we uncovered three previously unidentified *HER2* fusion genes in GC patients whose tumors were clinically classified as HER2-positive. Our results suggest that these *HER2* fusions are genetically amplified driver oncogenes that respond differently to the HER2-neutralizing antibody trastuzumab. The resistance of ZNF207-HER2 to trastuzumab and the existence of the recurrent *BRAF* fusion variants warrant molecular subtype diagnoses of HER*2-*positive GC patients for more effective personalized trastuzumab therapies and for future treatment options.

## Additional file

Additional file 1:
**Gene fusion CDS sequence of** ***ZNF207-HER2*** **fusion** ***MDK-HER2*** **fusion and** ***NOS2-HER2*** **fusion.** **File S1** ***.*** *ZNF207-HER2* gene fusion CDS sequence. **File S2.** *MDK-HER2* gene fusion CDS sequence. **File S3.** *NOS2-HER2* gene fusion CDS sequence.
