# Supplementary material for: Oncogenic HER2 fusions in gastric cancer
Source: J Transl Med. 2015 Apr 11;13:116. doi: 10.1186/s12967-015-0476-2 (PMC4396883; doi:10.1186/s12967-015-0476-2)
Supplement: Additional file 1: — Gene fusion CDS sequence of ZNF207-HER2 fusion MDK-HER2 fusion and NOS2-HER2 fusion. File S1 . ZNF207-HER2 gene fusion CDS sequence. File S2. MDK-HER2 gene fusion CDS sequence. File S3. NOS2-HER2 gene fusion CDS sequence. [file 12967_2015_476_MOESM1_ESM.docx]

**Additional file 1: Gene fusion CDS sequence of *ZNF207-HER2* fusion *MDK-HER2* fusion and *NOS2-HER2* fusion.**

**File S1*. ZNF207-HER2* gene fusion CDS sequence**

atgggtcgcaagaagaagaagcagctgaagccgtggtgctggtattgtaatagagattttgatgatgagaagatccttattcagcaccaaaaagcaaagcattttaaatgccatatatgtcacaagaaattgtatacaggacctggcttagctattcattgcatgca**gG**AGGCTGACCAGTGTGTGGCCTGTGCCCACTATAAGGACCCTCCCTTCTGCGTGGCCCGCTGCCCCAGCGGTGTGAAACCTGACCTCTCCTACATGCCCATCTGGAAGTTTCCAGATGAGGAGGGCGCATGCCAGCCTTGCCCCATCAACTGCACCCACTCCTGTGTGGACCTGGATGACAAGGGCTGCCCCGCCGAGCAGAGAGCCAGCCCTCTGACGTCCATCATCTCTGCGGTGGTTGGCATTCTGCTGGTCGTGGTCTTGGGGGTGGTCTTTGGGATCCTCATCAAGCGACGGCAGCAGAAGATCCGGAAGTACACGATGCGGAGACTGCTGCAGGAAACGGAGCTGGTGGAGCCGCTGACACCTAGCGGAGCGATGCCCAACCAGGCGCAGATGCGGATCCTGAAAGAGACGGAGCTGAGGAAGGTGAAGGTGCTTGGATCTGGCGCTTTTGGCACAGTCTACAAGGGCATCTGGATCCCTGATGGGGAGAATGTGAAAATTCCAGTGGCCATCAAAGTGTTGAGGGAAAACACATCCCCCAAAGCCAACAAAGAAATCTTAGACGAAGCATACGTGATGGCTGGTGTGGGCTCCCCATATGTCTCCCGCCTTCTGGGCATCTGCCTGACATCCACGGTGCAGCTGGTGACACAGCTTATGCCCTATGGCTGCCTCTTAGACCATGTCCGGGAAAACCGCGGACGCCTGGGCTCCCAGGACCTGCTGAACTGGTGTATGCAGATTGCCAAGGGGATGAGCTACCTGGAGGATGTGCGGCTCGTACACAGGGACTTGGCCGCTCGGAACGTGCTGGTCAAGAGTCCCAACCATGTCAAAATTACAGACTTCGGGCTGGCTCGGCTGCTGGACATTGACGAGACAGAGTACCATGCAGATGGGGGCAAGGTGCCCATCAAGTGGATGGCGCTGGAGTCCATTCTCCGCCGGCGGTTCACCCACCAGAGTGATGTGTGGAGTTATGGTGTGACTGTGTGGGAGCTGATGACTTTTGGGGCCAAACCTTACGATGGGATCCCAGCCCGGGAGATCCCTGACCTGCTGGAAAAGGGGGAGCGGCTGCCCCAGCCCCCCATCTGCACCATTGATGTCTACATGATCATGGTCAAATGTTGGATGATTGACTCTGAATGTCGGCCAAGATTCCGGGAGTTGGTGTCTGAATTCTCCCGCATGGCCAGGGACCCCCAGCGCTTTGTGGTCATCCAGAATGAGGACTTGGGCCCAGCCAGTCCCTTGGACAGCACCTTCTACCGCTCACTGCTGGAGGACGATGACATGGGGGACCTGGTGGATGCTGAGGAGTATCTGGTACCCCAGCAGGGCTTCTTCTGTCCAGACCCTGCCCCGGGCGCTGGGGGCATGGTCCACCACAGGCACCGCAGCTCATCTACCAGGAGTGGCGGTGGGGACCTGACACTAGGGCTGGAGCCCTCTGAAGAGGAGGCCCCCAGGTCTCCACTGGCACCCTCCGAAGGGGCTGGCTCCGATGTATTTGATGGTGACCTGGGAATGGGGGCAGCCAAGGGGCTGCAAAGCCTCCCCACACATGACCCCAGCCCTCTACAGCGGTACAGTGAGGACCCCACAGTACCCCTGCCCTCTGAGACTGATGGCTACGTTGCCCCCCTGACCTGCAGCCCCCAGCCTGAATATGTGAACCAGCCAGATGTTCGGCCCCAGCCCCCTTCGCCCCGAGAGGGCCCTCTGCCTGCTGCCCGACCTGCTGGTGCCACTCTGGAAAGGCCCAAGACTCTCTCCCCAGGGAAGAATGGGGTCGTCAAAGACGTTTTTGCCTTTGGGGGTGCCGTGGAGAACCCCGAGTACTTGACACCCCAGGGAGGAGCTGCCCCTCAGCCCCACCCTCCTCCTGCCTTCAGCCCAGCCTTCGACAACCTCTATTACTGGGACCAGGACCCACCAGAGCGGGGGGCTCCACCCAGCACCTTCAAAGGGACACCTACGGCAGAGAACCCAGAGTACCTGGGTCTGGACGTGCCAGTGTGA

**File S2. *MDK-HER2* gene fusion CDS sequence**

atgcagcaccgaggcttcctcctcctcaccctcctcgccctgctggcgctcacctccgcggtcgccaaaaagaaagataaggtgaagaagggcggcccggggagcgagtgcgctgagtgggcctgggggccctgcacccccagcagcaaggattgcggcgtgggtttccgcgagggcacctgcggggcccagacccagcgcatccggtgcagggtgccctgcaactggaagaaggagtttggagccgactgcaagtacaagtttgagaactggggtgcgtgtgatgggggcacaggcaccaaagtccgccaaggcaccctgaagaaggcgcgctacaatgctcagtgccaggagaccatccgcgtcaccaagccctgcacccccaagaccaaagcaaaggccaaa**gA**CAACTACCTTTCTACGGACGTGGGATCCTGCACCCTCGTCTGCCCCCTGCACAACCAAGAGGTGACAGCAGAGGATGGAACACAGCGGTGTGAGAAGTGCAGCAAGCCCTGTGCCCGAGTGTGCTATGGTCTGGGCATGGAGCACTTGCGAGAGGTGAGGGCAGTTACCAGTGCCAATATCCAGGAGTTTGCTGGCTGCAAGAAGATCTTTGGGAGCCTGGCATTTCTGCCGGAGAGCTTTGATGGGGACCCAGCCTCCAACACTGCCCCGCTCCAGCCAGAGCAGCTCCAAGTGTTTGAGACTCTGGAAGAGATCACAGGTTACCTATACATCTCAGCATGGCCGGACAGCCTGCCTGACCTCAGCGTCTTCCAGAACCTGCAAGTAATCCGGGGACGAATTCTGCACAATGGCGCCTACTCGCTGACCCTGCAAGGGCTGGGCATCAGCTGGCTGGGGCTGCGCTCACTGAGGGAACTGGGCAGTGGACTGGCCCTCATCCACCATAACACCCACCTCTGCTTCGTGCACACGGTGCCCTGGGACCAGCTCTTTCGGAACCCGCACCAAGCTCTGCTCCACACTGCCAACCGGCCAGAGGACGAGTGTGTGGGCGAGGGCCTGGCCTGCCACCAGCTGTGCGCCCGAGGGCACTGCTGGGGTCCAGGGCCCACCCAGTGTGTCAACTGCAGCCAGTTCCTTCGGGGCCAGGAGTGCGTGGAGGAATGCCGAGTACTGCAGGGGCTCCCCAGGGAGTATGTGAATGCCAGGCACTGTTTGCCGTGCCACCCTGAGTGTCAGCCCCAGAATGGCTCAGTGACCTGTTTTGGACCGGAGGCTGACCAGTGTGTGGCCTGTGCCCACTATAAGGACCCTCCCTTCTGCGTGGCCCGCTGCCCCAGCGGTGTGAAACCTGACCTCTCCTACATGCCCATCTGGAAGTTTCCAGATGAGGAGGGCGCATGCCAGCCTTGCCCCATCAACTGCACCCACTCCTGTGTGGACCTGGATGACAAGGGCTGCCCCGCCGAGCAGAGAGCCAGCCCTCTGACGTCCATCATCTCTGCGGTGGTTGGCATTCTGCTGGTCGTGGTCTTGGGGGTGGTCTTTGGGATCCTCATCAAGCGACGGCAGCAGAAGATCCGGAAGTACACGATGCGGAGACTGCTGCAGGAAACGGAGCTGGTGGAGCCGCTGACACCTAGCGGAGCGATGCCCAACCAGGCGCAGATGCGGATCCTGAAAGAGACGGAGCTGAGGAAGGTGAAGGTGCTTGGATCTGGCGCTTTTGGCACAGTCTACAAGGGCATCTGGATCCCTGATGGGGAGAATGTGAAAATTCCAGTGGCCATCAAAGTGTTGAGGGAAAACACATCCCCCAAAGCCAACAAAGAAATCTTAGACGAAGCATACGTGATGGCTGGTGTGGGCTCCCCATATGTCTCCCGCCTTCTGGGCATCTGCCTGACATCCACGGTGCAGCTGGTGACACAGCTTATGCCCTATGGCTGCCTCTTAGACCATGTCCGGGAAAACCGCGGACGCCTGGGCTCCCAGGACCTGCTGAACTGGTGTATGCAGATTGCCAAGGGGATGAGCTACCTGGAGGATGTGCGGCTCGTACACAGGGACTTGGCCGCTCGGAACGTGCTGGTCAAGAGTCCCAACCATGTCAAAATTACAGACTTCGGGCTGGCTCGGCTGCTGGACATTGACGAGACAGAGTACCATGCAGATGGGGGCAAGGTGCCCATCAAGTGGATGGCGCTGGAGTCCATTCTCCGCCGGCGGTTCACCCACCAGAGTGATGTGTGGAGTTATGGTGTGACTGTGTGGGAGCTGATGACTTTTGGGGCCAAACCTTACGATGGGATCCCAGCCCGGGAGATCCCTGACCTGCTGGAAAAGGGGGAGCGGCTGCCCCAGCCCCCCATCTGCACCATTGATGTCTACATGATCATGGTCAAATGTTGGATGATTGACTCTGAATGTCGGCCAAGATTCCGGGAGTTGGTGTCTGAATTCTCCCGCATGGCCAGGGACCCCCAGCGCTTTGTGGTCATCCAGAATGAGGACTTGGGCCCAGCCAGTCCCTTGGACAGCACCTTCTACCGCTCACTGCTGGAGGACGATGACATGGGGGACCTGGTGGATGCTGAGGAGTATCTGGTACCCCAGCAGGGCTTCTTCTGTCCAGACCCTGCCCCGGGCGCTGGGGGCATGGTCCACCACAGGCACCGCAGCTCATCTACCAGGAGTGGCGGTGGGGACCTGACACTAGGGCTGGAGCCCTCTGAAGAGGAGGCCCCCAGGTCTCCACTGGCACCCTCCGAAGGGGCTGGCTCCGATGTATTTGATGGTGACCTGGGAATGGGGGCAGCCAAGGGGCTGCAAAGCCTCCCCACACATGACCCCAGCCCTCTACAGCGGTACAGTGAGGACCCCACAGTACCCCTGCCCTCTGAGACTGATGGCTACGTTGCCCCCCTGACCTGCAGCCCCCAGCCTGAATATGTGAACCAGCCAGATGTTCGGCCCCAGCCCCCTTCGCCCCGAGAGGGCCCTCTGCCTGCTGCCCGACCTGCTGGTGCCACTCTGGAAAGGCCCAAGACTCTCTCCCCAGGGAAGAATGGGGTCGTCAAAGACGTTTTTGCCTTTGGGGGTGCCGTGGAGAACCCCGAGTACTTGACACCCCAGGGAGGAGCTGCCCCTCAGCCCCACCCTCCTCCTGCCTTCAGCCCAGCCTTCGACAACCTCTATTACTGGGACCAGGACCCACCAGAGCGGGGGGCTCCACCCAGCACCTTCAAAGGGACACCTACGGCAGAGAACCCAGAGTACCTGGGTCTGGACGTGCCAGTGTGA

**File S3. *NOS2-HER2* gene fusion CDS sequence**

atggcctgtccttggaaatttctgttcaagaccaaattccaccagtatgcaatgaatggggaaaaagacatcaacaacaatgtggagaaagccccctgtgccacctcca**gT**CGCAATTGAAGTACCACCTCCCGAGGGTGATTGCTTCCCCATGCGGGGTAGAACCTTTGCTGTCCTGTTCACCACTCTACCTCCAGCACAGAATTTGGCTTATGCCTACTCAATGTGAAGATGATGAGGATGAAAACCTTTGTGATGATCCACTTCCACTTAATGAATGGTGGCAAAGCAAAGCTATATTCAAGACCACATGCAAAGCTACTCCCTGAGCAAAGAGTCACAGATAAAACGGGGGCACCAGTAGAATGGCCAGGACAAACGCAGTGCAGCACAGAGACTCAGACCCTGGCAGCCATGCCTGCGCAGGCAGTGATGAGAGTGACATGTACTGTTGTGGACATGCACAAAAGTGAGTGTGCACCGGCACAGACATGAAGCTGCGGCTCCCTGCCAGTCCCGAGACCCACCTGGACATGCTCCGCCACCTCTACCAGGGCTGCCAGGTGGTGCAGGGAAACCTGGAACTCACCTACCTGCCCACCAATGCCAGCCTGTCCTTCCTGCAGGATATCCAGGAGGTGCAGGGCTACGTGCTCATCGCTCACAACCAAGTGAGGCAGGTCCCACTGCAGAGGCTGCGGATTGTGCGAGGCACCCAGCTCTTTGAGGACAACTATGCCCTGGCCGTGCTAGACAATGGAGACCCGCTGAACAATACCACCCCTGTCACAGGGGCCTCCCCAGGAGGCCTGCGGGAGCTGCAGCTTCGAAGCCTCACAGAGATCTTGAAAGGAGGGGTCTTGATCCAGCGGAACCCCCAGCTCTGCTACCAGGACACGATTTTGTGGAAGGACATCTTCCACAAGAACAACCAGCTGGCTCTCACACTGATAGACACCAACCGCTCTCGGGCCTGCCACCCCTGTTCTCCGATGTGTAAGGGCTCCCGCTGCTGGGGAGAGAGTTCTGAGGATTGTCAGAGCCTGACGCGCACTGTCTGTGCCGGTGGCTGTGCCCGCTGCAAGGGGCCACTGCCCACTGACTGCTGCCATGAGCAGTGTGCTGCCGGCTGCACGGGCCCCAAGCACTCTGACTGCCTGGCCTGCCTCCACTTCAACCACAGTGGCATCTGTGAGCTGCACTGCCCAGCCCTGGTCACCTACAACACAGACACGTTTGAGTCCATGCCCAATCCCGAGGGCCGGTATACATTCGGCGCCAGCTGTGTGACTGCCTGTCCCTACAACTACCTTTCTACGGACGTGGGATCCTGCACCCTCGTCTGCCCCCTGCACAACCAAGAGGTGACAGCAGAGGATGGAACACAGCGGTGTGAGAAGTGCAGCAAGCCCTGTGCCCGAGTGTGCTATGGTCTGGGCATGGAGCACTTGCGAGAGGTGAGGGCAGTTACCAGTGCCAATATCCAGGAGTTTGCTGGCTGCAAGAAGATCTTTGGGAGCCTGGCATTTCTGCCGGAGAGCTTTGATGGGGACCCAGCCTCCAACACTGCCCCGCTCCAGCCAGAGCAGCTCCAAGTGTTTGAGACTCTGGAAGAGATCACAGGTTACCTATACATCTCAGCATGGCCGGACAGCCTGCCTGACCTCAGCGTCTTCCAGAACCTGCAAGTAATCCGGGGACGAATTCTGCACAATGGCGCCTACTCGCTGACCCTGCAAGGGCTGGGCATCAGCTGGCTGGGGCTGCGCTCACTGAGGGAACTGGGCAGTGGACTGGCCCTCATCCACCATAACACCCACCTCTGCTTCGTGCACACGGTGCCCTGGGACCAGCTCTTTCGGAACCCGCACCAAGCTCTGCTCCACACTGCCAACCGGCCAGAGGACGAGTGTGTGGGCGAGGGCCTGGCCTGCCACCAGCTGTGCGCCCGAGGGCACTGCTGGGGTCCAGGGCCCACCCAGTGTGTCAACTGCAGCCAGTTCCTTCGGGGCCAGGAGTGCGTGGAGGAATGCCGAGTACTGCAGGGGCTCCCCAGGGAGTATGTGAATGCCAGGCACTGTTTGCCGTGCCACCCTGAGTGTCAGCCCCAGAATGGCTCAGTGACCTGTTTTGGACCGGAGGCTGACCAGTGTGTGGCCTGTGCCCACTATAAGGACCCTCCCTTCTGCGTGGCCCGCTGCCCCAGCGGTGTGAAACCTGACCTCTCCTACATGCCCATCTGGAAGTTTCCAGATGAGGAGGGCGCATGCCAGCCTTGCCCCATCAACTGCACCCACTCCTGTGTGGACCTGGATGACAAGGGCTGCCCCGCCGAGCAGAGAGCCAGCCCTCTGACGTCCATCATCTCTGCGGTGGTTGGCATTCTGCTGGTCGTGGTCTTGGGGGTGGTCTTTGGGATCCTCATCAAGCGACGGCAGCAGAAGATCCGGAAGTACACGATGCGGAGACTGCTGCAGGAAACGGAGCTGGTGGAGCCGCTGACACCTAGCGGAGCGATGCCCAACCAGGCGCAGATGCGGATCCTGAAAGAGACGGAGCTGAGGAAGGTGAAGGTGCTTGGATCTGGCGCTTTTGGCACAGTCTACAAGGGCATCTGGATCCCTGATGGGGAGAATGTGAAAATTCCAGTGGCCATCAAAGTGTTGAGGGAAAACACATCCCCCAAAGCCAACAAAGAAATCTTAGACGAAGCATACGTGATGGCTGGTGTGGGCTCCCCATATGTCTCCCGCCTTCTGGGCATCTGCCTGACATCCACGGTGCAGCTGGTGACACAGCTTATGCCCTATGGCTGCCTCTTAGACCATGTCCGGGAAAACCGCGGACGCCTGGGCTCCCAGGACCTGCTGAACTGGTGTATGCAGATTGCCAAGGGGATGAGCTACCTGGAGGATGTGCGGCTCGTACACAGGGACTTGGCCGCTCGGAACGTGCTGGTCAAGAGTCCCAACCATGTCAAAATTACAGACTTCGGGCTGGCTCGGCTGCTGGACATTGACGAGACAGAGTACCATGCAGATGGGGGCAAGGTGCCCATCAAGTGGATGGCGCTGGAGTCCATTCTCCGCCGGCGGTTCACCCACCAGAGTGATGTGTGGAGTTATGGTGTGACTGTGTGGGAGCTGATGACTTTTGGGGCCAAACCTTACGATGGGATCCCAGCCCGGGAGATCCCTGACCTGCTGGAAAAGGGGGAGCGGCTGCCCCAGCCCCCCATCTGCACCATTGATGTCTACATGATCATGGTCAAATGTTGGATGATTGACTCTGAATGTCGGCCAAGATTCCGGGAGTTGGTGTCTGAATTCTCCCGCATGGCCAGGGACCCCCAGCGCTTTGTGGTCATCCAGAATGAGGACTTGGGCCCAGCCAGTCCCTTGGACAGCACCTTCTACCGCTCACTGCTGGAGGACGATGACATGGGGGACCTGGTGGATGCTGAGGAGTATCTGGTACCCCAGCAGGGCTTCTTCTGTCCAGACCCTGCCCCGGGCGCTGGGGGCATGGTCCACCACAGGCACCGCAGCTCATCTACCAGGAGTGGCGGTGGGGACCTGACACTAGGGCTGGAGCCCTCTGAAGAGGAGGCCCCCAGGTCTCCACTGGCACCCTCCGAAGGGGCTGGCTCCGATGTATTTGATGGTGACCTGGGAATGGGGGCAGCCAAGGGGCTGCAAAGCCTCCCCACACATGACCCCAGCCCTCTACAGCGGTACAGTGAGGACCCCACAGTACCCCTGCCCTCTGAGACTGATGGCTACGTTGCCCCCCTGACCTGCAGCCCCCAGCCTGAATATGTGAACCAGCCAGATGTTCGGCCCCAGCCCCCTTCGCCCCGAGAGGGCCCTCTGCCTGCTGCCCGACCTGCTGGTGCCACTCTGGAAAGGCCCAAGACTCTCTCCCCAGGGAAGAATGGGGTCGTCAAAGACGTTTTTGCCTTTGGGGGTGCCGTGGAGAACCCCGAGTACTTGACACCCCAGGGAGGAGCTGCCCCTCAGCCCCACCCTCCTCCTGCCTTCAGCCCAGCCTTCGACAACCTCTATTACTGGGACCAGGACCCACCAGAGCGGGGGGCTCCACCCAGCACCTTCAAAGGGACACCTACGGCAGAGAACCCAGAGTACCTGGGTCTGGACGTGCCAGTGTGA
